# Seasonal Trends in the Abundance of Eurasian Woodcock Populations Monitored Using “Beccapp” in Apulia, and the Age Structure of Shot Birds

**DOI:** 10.3390/ani16101442

**Published:** 2026-05-08

**Authors:** Simona Tarricone, Marco Tuti, Paolo Pennacchini, Maria Antonietta Colonna, Giuseppe La Gioia, Domenico Campanile, Maria Teresa Carone, Giuseppe Raho, Marco Ragni

**Affiliations:** 1 Department of Soil, Plant and Food Sciences, University of Bari Aldo Moro, 70126 Bari, Italy; simona.tarricone@uniba.it (S.T.); marco.ragni@uniba.it (M.R.); 2 FANBPO, “Fédération des Associations Nationales des Bécassiers du Paléarctique Occidental” and FIBec, “Federazione Italiana Beccacciai”, Via Fausto Vagnetti 12, 52031 Anghiari, Italy; p.pennacchini@fanbpo.com (P.P.); giusepperaho@gmail.com (G.R.); 3 Or.Me, 73100 Lecce, Italy; lagioiagiu@gmail.com; 4 Regione Puglia, Dipartimento Agricoltura, Sviluppo Rurale ed Ambientale, Sezione Gestione Sostenibile e Tutela delle Risorse Forestali e Naturali, 70124 Bari, Italy; d.campanile@regione.puglia.it (D.C.); mt.carone@regione.puglia.it (M.T.C.); 5 Centro Studi Beccaccia Puglia (CSB Puglia), Via Clemente Rebora 14, 73100 Lecce, Italy

**Keywords:** monitoring, “Beccapp” application, demographic structure, age classes, migration, wing collection

## Abstract

Monitoring population trends of the Eurasian Woodcock (*Scolopax rusticola*) is challenging due to its cryptic behaviour and migratory dynamics. In this study, we analysed hunting and post-hunting data collected through the “Beccapp” application to assess temporal patterns of the abundance and demographic structure of woodcock in the Apulia region (southern Italy) over three consecutive seasons (2022/2023, 2023/2024 and 2024/2025). A total of 2580 trips were recorded, including 1795 hunting trips and 785 post-hunting monitoring trips, covering 89 municipalities. Relative abundance was estimated using the Index of Abundance per Trip (ICA). Mean ICA values remained stable across seasons and were highly correlated when calculated using all surveyed municipalities or only those consistently monitored (Pearson’s r = 0.998, *p* < 0.001), indicating robustness to spatial variability. Decadal analyses revealed two main ICA peaks (0.86 in late November–early December and 1.1 in late February–early March), corresponding to post-nuptial and pre-nuptial migration periods. Demographic analyses based on wing collections showed a predominance of juveniles (83.2%) in the hunting bag, with no significant differences in body mass between age classes. These results highlight the value of standardized hunter-based monitoring for monitoring the abundance of Eurasian Woodcock and supporting sustainable management.

## Simple Summary

The Eurasian Woodcock is a migratory forest bird that is difficult to monitor using traditional survey methods. In this study, we used data collected by hunters through a smartphone application to monitor woodcock presence in Apulia region, southern Italy, over three consecutive monitoring seasons. Both hunting trips and non-hunting monitoring trips were analysed to describe changes in woodcock abundance over time. The results showed that woodcock abundance remained stable across years and followed clear seasonal patterns, with peaks during autumn and late winter migration periods. Most hunted birds were juveniles, while no differences in body weight were found between age classes. Our findings show that hunter-based data collection can provide relevant information on the distribution and abundance of woodcock and on the age structure of birds that are shot, supporting the informed and sustainable management of woodcock populations.

## 1. Introduction

The Eurasian Woodcock *Scolopax rusticola* is a migratory and solitary wader that inhabits forested environments. Its breeding range extends across central, northern and eastern Europe, while during winter it mainly occupies areas in western and southern Europe, including France, the British Isles, the Iberian Peninsula, Italy and regions surrounding the Mediterranean basin [1,2]. As with many other waders belonging to the order *Charadrii*, the species exhibits sexual monomorphism, with males and females showing very similar plumage characteristics and body size, although minor differences between sexes have been reported [1,3,4].

The Eurasian Woodcock is widely hunted and represents a valued game species in many European countries [5]. The European population is estimated at approximately 13.8–17.4 million adults and is currently considered stable [6]. For this reason, it is listed under Annex II/A of the EU Birds Directive [7], which allows regulated hunting throughout the European Union. Harvesting generally occurs during autumn and winter across most of its range [8]. Hunting pressure is particularly high in France and Italy, where woodcock meat is traditionally appreciated [9,10]. In Italy, the hunting season usually begins in the third week of September and ends on 31 January, although in certain regions it may end earlier, on 31 December. Despite its importance as a woody species, the impact of hunting on annual population dynamics and long-term abundance remains poorly understood. In addition to hunting pressure, habitat loss and fragmentation may further influence population trends. Game management practices are typically implemented at local or national scales; however, for migratory species such as the Eurasian Woodcock, effective management requires coordination among all countries involved along the migratory route [11]. Understanding migratory connectivity between breeding, stopover, and wintering areas is therefore essential to identify regions where management strategies should be harmonized. The sustainable exploitation of game species depends on adjusting harvest levels in response to fluctuations in population size, for example, by reducing hunting pressure in years characterized by low reproductive success or increased natural mortality [12]. Consequently, identifying the factors that influence population abundance is fundamental for informed decision-making in wildlife management [13]. Recent advances in tracking and remote-sensing technologies have substantially improved our capacity to study Eurasian Woodcock ecology and migratory behaviour. The deployment of miniaturized GPS and GPS-GSM transmitters has enabled fine-scale reconstruction of individual migration routes, stopover site selection, and wintering area fidelity, revealing a high degree of individual variation in migratory strategy that was previously undetectable through ringing alone [14,15]. Light-level geolocators, despite their lower spatial resolution, have similarly contributed to flyway-scale descriptions of connectivity between breeding and wintering populations across Europe [16]. More recently, the expansion of automated radio-telemetry networks—such as the Motus Wildlife Tracking System—has allowed passive, large-scale monitoring of individually tagged birds at low logistical cost, offering new possibilities for tracking nocturnal migrants like the Eurasian Woodcock across broad geographic areas [17]. Alongside these individual-based approaches, acoustic monitoring and thermal imaging have been explored as non-invasive survey tools, while citizen science platforms and hunter-reporting applications are increasingly integrated into national and European monitoring frameworks [18]. Together, these developments have highlighted the complexity of woodcock migration and the need for monitoring approaches that can operate at multiple spatial and temporal scales. Standardized hunter-based monitoring, in particular, remains indispensable at the regional level: its broad coverage, cost-effectiveness, and continuity over time make it complementary to the finer but spatially limited insights provided by telemetry and banding studies [19].

Monitoring population size and incorporating demographic parameters into population models are key components of adaptive management strategies. Abundance can be assessed either through direct methods, such as density estimates, or by using indirect indices [20,21], both of which are commonly applied to hunted species. Several indices have been developed to estimate the abundance of game bird populations, including information derived from hunter-collected data during the hunting season [4] and those obtained through standardized nocturnal banding surveys [22].

The monitoring of Eurasian Woodcock populations is essential for estimating abundance and for examining relationships with climatic, environmental, and anthropogenic drivers, thereby supporting evidence-based management strategies. Abundance indices are widely used in ecological research and monitoring frameworks because they are straightforward to compute, easy to interpret, and supported by a long record of application [19]. Indices that quantify abundance as the average number of individuals recorded at occupied sites are generally considered more informative than occurrence-based indices, which typically rely on scaled measures of site occupancy relative to observed birds [23]. Many researchers have employed abundance indices to investigate population densities of wild bird assemblages, providing insights into their spatial patterns [24,25], habitat associations, species diversity, and responses to local environmental variability [26,27].

Hence, the Woodcock population in Italy has been categorized as “Least Concern” in 2021, reflecting a secure conservation status [18,22]. However, to date no population estimates have been carried out for the Apulian region [28]. Thus, this study aims to (i) provide a preliminary estimate of the abundance of the species in the region during the period 2022–2025 by collecting data through the “BECAPP” application, and (ii) analyse the demographic structure of the Scolopax population in Apulia through wing analysis.

## 2. Materials and Methods

A structured monitoring program was designed in collaboration with woodcock hunters in the Apulia region (south-eastern Italy) that lasted for 3 consecutive hunting seasons (2022–2023, 2023–2024 and 2024–2025) and the subsequent winter months, covering the period from September to March (Apulia Region authorization n. 51/2022; 64/2023; 46/2024). Hunters were trained in order to properly record information about both their hunts and the birds they caught. The monitoring program included two main components: (i) the estimation of abundance indices, derived from data collected during hunting and monitoring trips; (ii) the collection and analysis of wings from hunted woodcocks.

Participating hunters attended certified training courses aimed at providing the theoretical and practical skills necessary for standardized data collection during hunting and monitoring activities.

### 2.1. Abundance Indices and Hunting Effort

Woodcock abundance indices were estimated through standardized field observations. The training courses attended by hunters emphasized appropriate behavioural protocols during monitoring sessions, such as immediately stopping dogs after flushing a woodcock, to minimize disturbance to both target and non-target species. These courses were officially recognized by the Italian Ministry of Agriculture, Food Sovereignty and Forests and included practical demonstrations of data collection procedures, database entry, and a final exam to verify the participants’ skills. Upon completion of the training, observers were deemed adequately qualified to reliably detect and count woodcocks. All observations were uploaded into an online database (Beccapp) [29], accessible through individual user accounts. Since in Apulia woodcock hunting is allowed from 1 October to 20 January, two monitoring periods were distinguished in each year: the hunting period (1 October–20 January) and the post-hunting monitoring period (21 January–31 March). Consequently, we differentiated the hunts conducted to monitor the presence of woodcock in “hunting trips” conducted during the hunting season and “monitoring trips” carried out after the end of hunting season.

For each type of trip, observers recorded the date, municipality, duration of activity (hours and minutes), number of woodcocks flushed (estimated as different birds), number of participants, number of pointing dogs used, air temperature (recorded via smartphone), altitude class (500 m bands, recorded via smartphone), and vegetation type (deciduous woodland, coniferous woodland, or scrubland). To reduce the risk of overcounting birds that might have been flushed multiple times, hunters were instructed to record only woodcocks that flew away from the observer immediately after being flushed. All data are automatically saved in excel file, where they are filtered to check any writing errors, especially about date and duration. We do not consider trips with duration less than half hour and date out of the correct period.

These data were used to calculate a hunting index of abundance (ICA, Indice Cynégétique d’Abondance), defined as the number of different woodcocks flushed per standardized hunting effort. According to Tuti et al. [19], ICA was standardized to a reference duration of 3.5 h, representing the average length of a woodcock hunting trip in Europe, and adjusted for the number of hunters and pointing dogs involved. The index was calculated as

(1) $$\mathrm{ICA}=\left(\frac{\mathrm{number}\;\mathrm{of}\;\mathrm{woodcock}s\;\mathrm{flushed}}{\mathrm{trip}\;\mathrm{duration}\times \mathrm{number}\;\mathrm{of}\;\mathrm{participants}\times \mathrm{number}\;\mathrm{of}\;\mathrm{dogs}}\right)\times 3.5$$

During the hunting season, trips were conducted in accordance with Italian national law [30], which allows hunting activities five days per week (excluding Tuesdays and Fridays). During the post-hunting period (from 21 January, that is the official hunting closure date, to 31 March), monitoring trips were carried out on the same days. In both periods, surveys focused on huntable areas, predominantly forested habitats. Obviously, the area was an optional choice of the hunter, who was free to space out in his hunting district. It was the same for monitoring period too, so the probabilistic criteria of contacting woodcocks were the same. Anyway, given the differences in disturbance and bird removal between hunting and post-hunting periods, analyses were conducted separately for each phase.

The variation in woodcock abundance was evaluated during decadal periods (per 10-day period) and between seasons.

In addition to ICA, two further indices were calculated [31]:

(2) $$\mathrm{Hunting}\;\mathrm{mortality}\;\mathrm{rate}\;(\mathrm{IMC})\;=\;\left(\frac{\mathrm{number}\;\mathrm{of}\;\mathrm{woodcocks}\;\mathrm{killed}}{\mathrm{number}\;\mathrm{of}\;\mathrm{woodcocks}\;\mathrm{flushed}}\right)\times 100$$

Hunting effort (SdC), defined as the total duration of hunting trips divided by the number of woodcocks hunted, representing the average time required to hunt one bird.

### 2.2. Wing Collection and Demographic Analyses

Hunting data were obtained through the examination of wings collected by trained hunters; this study is a well-established approach for assessing seasonal variation in the abundance of migratory birds [32,33] and for estimating population parameters such as age ratios [34]. Age ratios were determined using the analysis of dried wings following a protocol developed by the Club National des Bécassiers (CNB), which has proven to be highly reliable. Specifically, we applied the Boidot method [35], which considers not only primary flight feathers [3,4,36] but also greater primary and secondary coverts [4,35]. This method was introduced in Italy in 2004 by the Associazione Beccacciai d’Italia (currently FIBec, Federazione Italiana Beccacciai) through the “Ali d’Italia” project.

The demographic structure of *Scolopax rusticola* in Apulia was assessed using wing samples collected during two hunting seasons, i.e., 2022–2023 and 2024–2025. Hunters using pointing dogs were supplied with envelopes for sample collection. The right wing of each hunted bird was put into an envelope, after completing an attached data sheet reporting the date and municipality of hunting and body mass (measured to the nearest gram using a digital scale). At the end of each season, all envelopes were retrieved and submitted to certified FIBec readers for analysis.

Hunting data were examined on a ten-day (decadal) basis to infer migration phenology and wintering dynamics. Demographic classification relied on feather wear patterns and moult suspension. Woodcock interrupt moult prior to post-breeding migration; therefore, during the Apulian hunting season (1 October–20 January), the number of unmoulted feathers remains stable. Juveniles and adults were initially distinguished by evaluating feather wear, colour, and shape on the external wing surface. Juvenile feathers are typically more worn, duller, and show pointed, frayed tips. Juvenile age was further refined based on the number of unmoulted secondary greater coverts and alula feathers, which increases with later hatching dates. Juveniles were subsequently assigned to five birth-month classes (Jc0: April; Jc1: May; Jc2: June; Jc3: July; Jc4: August). In adults, the internal wing surface was examined to identify birds with completed moult (Ac0), one-year-old birds retaining some juvenile feathers (An + 1), and birds aged two years or older (An + x), in which all feathers are of adult type [4,35]. From the data collected, age ratio (juveniles/adults) and body mass, analysed both as seasonal averages and as decadal trends, were calculated.

### 2.3. Statistical Analysis

All statistical analyses were performed in R version 4.5.2 (R Core Team, Vienna, Austria, 2025) [37]. To evaluate the temporal trend of the Index of Abundance per Trip (ICA) across years, we calculated the mean ICA value for each hunting season (1 October–20 January). Because the set of municipalities varied slightly among seasons, the robustness of the mean ICA was assessed by comparing it with the mean ICA calculated using only the municipalities surveyed consistently across all three seasons. The relationship between the two ICA series was tested using Pearson’s correlation coefficient.

Hunting and post-hunting data were analysed separately. Hunting and monitoring effort (number of trips, hours, observations, and harvested individuals) was aggregated by decade (10-day periods). Decadal ICA values were calculated as the mean of ICA values from individual trips occurring within each decade, and variability was expressed as standard deviation.

For demographic analyses, collected wings were classified by age class (juvenile vs. adult). Differences in mean body mass between juveniles and adults were assessed using Student’s *t*-tests for independent samples, after checking the assumptions of normality and homoscedasticity. For all statistical tests, significance was set at *p* < 0.05.

## 3. Results

### 3.1. Abundance Indices and Hunting Effort

Through the data collected using the “Beccapp” application, a total of 2580 trips recorded were used for the analyses. These trips were carried out by 78 hunters in the first season, 53 in the second, and 53 in the third season. From the 2022–2023 hunting season to the 2024–2025 hunting season, during the hunting period (1 October–20 January), 1795 woodcock hunting trips with pointing dogs were recorded. During the post-hunting period (21 January–31 March), 785 woodcock monitoring trips (without shooting) were conducted, for a total of 89 municipalities involved in at least one hunting or monitoring trip across all provinces. In total, 2805 woodcocks were observed during the hunting periods and 903 during the monitoring periods. Detailed information on hunting effort, number of observations, harvest, and abundance indices for each season is reported in Table 1.

**Figure 1:**
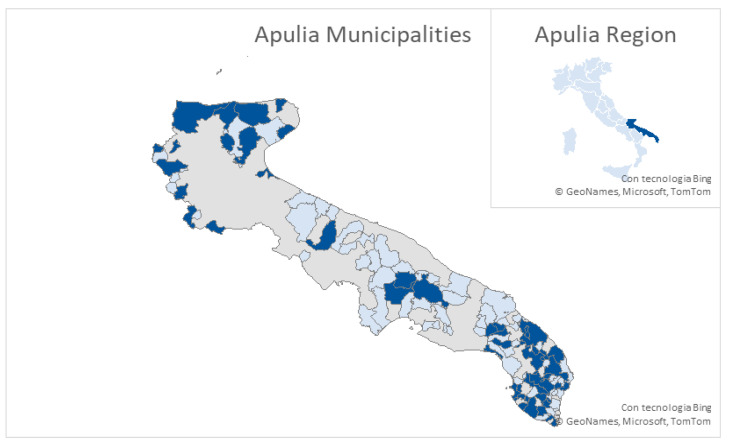
The map in the right-hand corner shows the position of the Apulia region in Italy. In the main map, the municipalities that were repeated every season are colored in dark blue, the municipalities visited only one year are in light blue, while all the other municipalities are grey.

Among the 89 municipalities considered, 39 were surveyed in all three seasons; these municipalities are shown in Figure 1.

**Figure 2:**
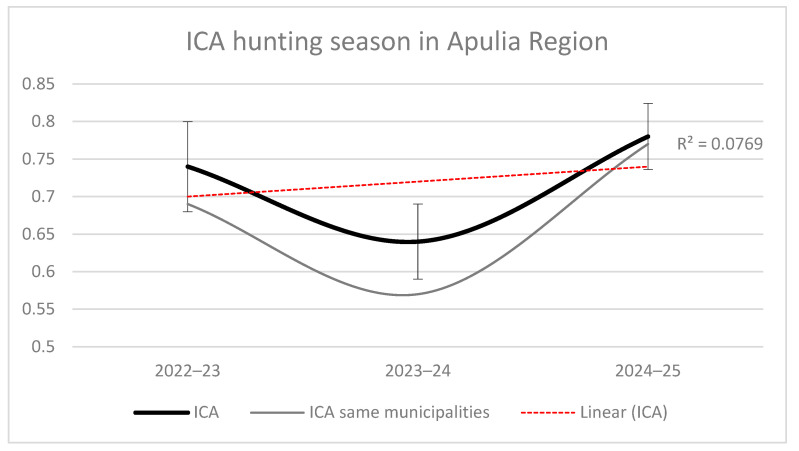
ICA trend between the three seasons: The black curve considers all the municipalities investigated and the bars indicate the 95% confidence interval. The gray curve, instead, represents the ICA trend considering only the municipalities that are the same season by season; the dotted red line shows the tendency of ICA over the time period.

Figure 2 shows the ICA trend across the three seasons. The black curve represents the ICA calculated using all municipalities surveyed in each season, with bars indicating the 95% confidence intervals. The grey curve represents the ICA trend calculated using only the municipalities common to all seasons, while the dotted red line indicates the temporal trend. The Pearson correlation analysis revealed a strong and significant positive relationship between the two ICA series (r = 0.998, *p* < 0.001; Table 2), indicating that variations in spatial coverage among seasons did not substantially affect the overall ICA trend. The ICA remained stable over the three-year period, as also indicated by the high coefficient of determination (r^2^; Figure 2).

Data were subsequently aggregated and summarized by decade (10-day periods). As previously described, hunting and post-hunting periods were analysed separately due to differences in disturbance, bird removal, and hunting behaviour. During the monitoring period, trips were generally shorter, as hunters spent less time following individual birds and were overall less motivated. This resulted in higher ICA values during the February–March period (Table 1).

Most hunting and monitoring efforts occurred between November and early March, corresponding to the period when woodcocks are expected to be most abundant in the study area and reflecting their presence during migration and wintering.

**Figure 3:**
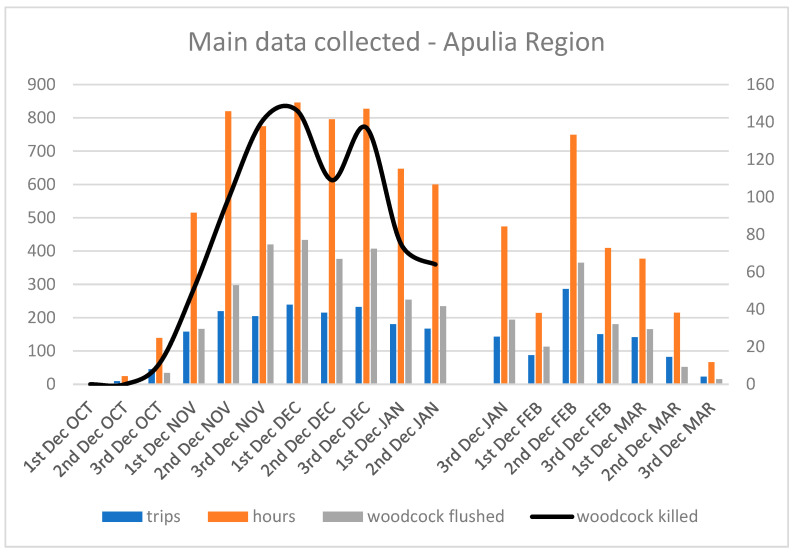
Summary data (trips, hours, contacts and killing) divided per decade during the entire monitoring period.

**Figure 4:**
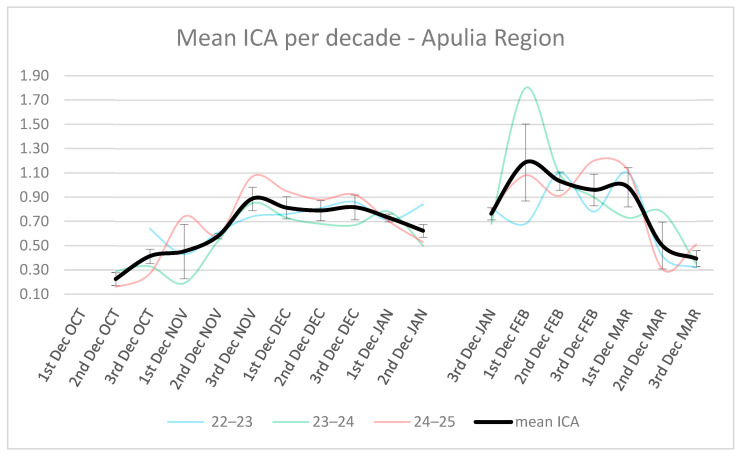
Mean ICA trend per decade (calculated as average between ICA value of each trip included in a period of ten days)—colored lines represent ICA per decade of each single season, while black line represents the mean between the three monitoring seasons; the bars indicate the Standard Deviation.

Figure 3 summarizes hunting and monitoring efforts (number of trips, hours, contacts, and hunted birds), by decade, across the whole monitoring period. The ICA curves (Figure 4) show the highest values between the third decade of November and the first decade of March. The decades with maximum ICA values likely correspond to periods of highest migratory influx, during which woodcock contingents use the Apulia region as a stopover site.

Overall, increases in ICA—particularly during the hunting period despite bird removal—are indicative of post-nuptial migration increase, followed by a more stable, but slightly decreasing, wintering phase. A second increase occurs during the pre-nuptial migration, when birds migrate in the opposite direction and temporarily stop in the area.

Figure 4 shows the temporal trend of mean ICA values across successive decadal (10-day) periods in the Apulia region. When averaged across the three monitoring seasons, mean ICA values increase from low levels in early October to higher values in November and December (ICA = 0.86), reaching a first plateau during late autumn. A marked increase is then observed in late January to early February (ICA = 1.10), followed by a gradual decline towards March (ICA < 0.40).

Inter-annual variability is evident, as indicated by the divergence among the coloured lines representing individual seasons, particularly during winter and late winter periods. Despite this variability, the overall mean trend (black line) highlights a consistent seasonal pattern, with ICA values increasing in mid-winter and decreasing during the pre-nuptial period. Standard deviation bars indicate moderate variability within decades, with larger dispersion observed during periods of rapid change in ICA values.

### 3.2. Wing Collection and Demographic Analyses

Table 3 shows the results of data collected in the 2022/2023 and 2024/2025 hunting seasons. A total of 277 and 437 wings were collected during 2022/2023 and 2024/2025, respectively, while no wing data were available for the 2023/2024 season. Each wing corresponded to a single hunted woodcock. Wings were classified by age (young vs adult). Young birds represented 83.19% of the total sample, indicating the predominantly presence of young birds in the Apulian woods. No significant difference in mean body weight was found between adults and juveniles (*p* = 0.543).

**Figure 5:**
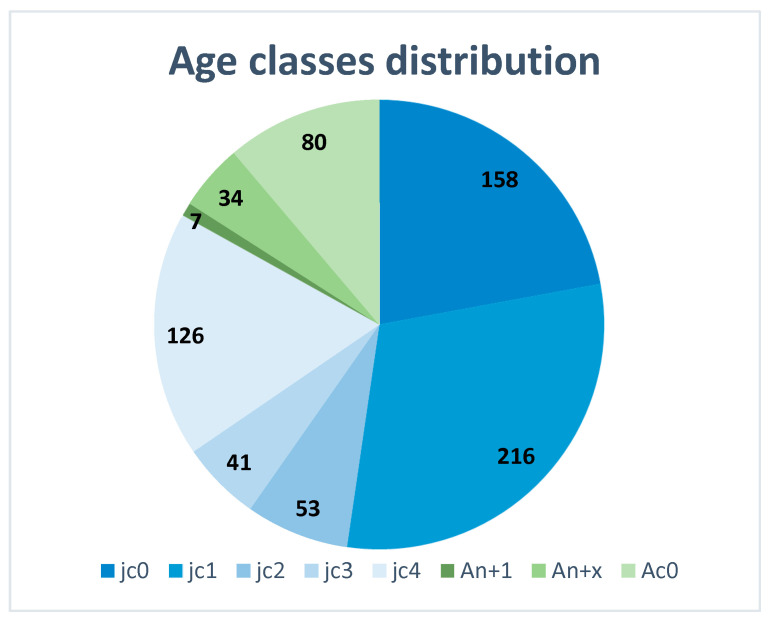
The distribution of *Scolopax rusticola* age classes: Jc0: April; Jc1: May; Jc2: June; Jc3: July; Jc4: August; An + 1: one-year-old birds retaining some juvenile feathers; An + x: birds aged two years or older; Ac0: individuals with completed moult.

The distribution of birds among age classes is reported in Figure 5. Notably, a relatively high number of birds classified as jc4 (juveniles hatched late in the season, July–August) was observed.

**Figure 6:**
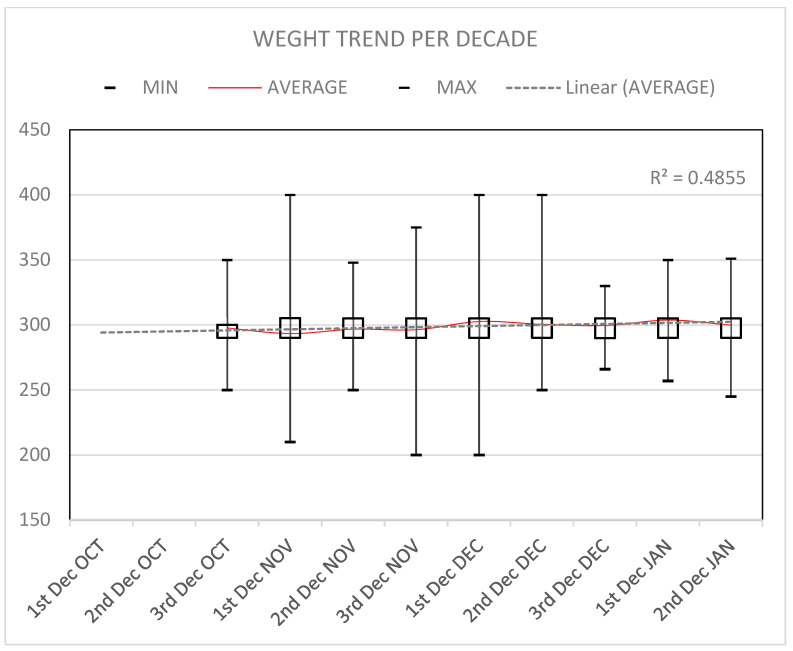
Weight variation during the hunting season monitored for each decade (red line): the bars indicate the range of variation (minimum and maximum), while the boxes the first and third quartiles; the dotted line indicates the tendence of the mean weight.

Figure 6 shows the variation in the body mass of Eurasian Woodcock across successive decadal (10-day) periods. Mean body mass shows an overall increasing trend from October to January, as indicated by the positive linear regression (r^2^ = 0.49). Average values increase from approximately 295–298 g in early October to peak values exceeding 300 g during December and early January. Despite short-term fluctuations between consecutive decades, particularly in late October and mid-December, the general pattern is characterized by a progressive increase in body mass over the study period.

## 4. Discussion

This study provides a three-year assessment of woodcock abundance, hunting effort, and demographic structure in the Apulian region, based on standardized hunter-collected data. Similar approaches integrating hunting and monitoring data have been successfully applied to woodcock and other migratory game birds across Europe, demonstrating their value for large-scale population monitoring [4,38]. While this framework offers broad spatial and temporal coverage at reasonable cost, a critical appraisal of its inherent limitations is necessary to properly contextualize the results presented here.

The Index of Abundance per Trip (ICA) proved to be a robust indicator of woodcock presence over time. The strong correlation between ICA values calculated using all surveyed municipalities and those derived from municipalities consistently monitored across seasons indicates that variations in spatial coverage did not bias the observed trends. This result is in line with previous studies showing that relative abundance indices based on standardized hunting effort can reliably track population dynamics when effort and methodology are controlled [4,39]. Nevertheless, it should be acknowledged that ICA values reflect detectability under hunter-specific conditions rather than absolute population density. Hunters do not distribute effort uniformly across the landscape; they preferentially target areas perceived as productive, accessible, and historically rewarding. This leads to a systematic overrepresentation of certain habitat types—particularly woodland edges, agricultural margins, and areas near roads—while remote or less accessible habitats receive disproportionately less effort. As a consequence, ICA values may reflect local woodcock abundance in hunter-preferred microhabitats rather than true regional population density, a persistent spatial bias that is difficult to fully correct for even when effort is standardized by trip.

The overall stability of ICA values across the three hunting seasons suggests the absence of major interannual fluctuations in woodcock abundance in the study area. Similar stability has been reported in other Mediterranean wintering areas, where local abundance is largely driven by migratory fluxes rather than by local demographic processes alone [40,41]. This supports the role of southern Italy as a regular wintering and stopover area along the central–eastern Mediterranean flyway [42]. Italy should be considered an important stopover and wintering area, due to its central position within the western Mediterranean basin. Within this framework, Apulia, located in south-eastern Italy, may host migrating birds that use the region as a temporary staging area to rest and refuel during both autumn and spring migration periods [43]. At a finer, regional scale, however, short-distance or local movements oriented along the Italian peninsula cannot be excluded and may reflect responses to local environmental conditions or habitat availability [42]. This apparent stability should nonetheless be interpreted with caution. Temporal selectivity in hunting effort is a well-recognised source of bias: hunters tend to concentrate activity during periods of expected high abundance, such as migration peaks, and reduce effort during poor weather or low perceived bird availability. This non-random temporal distribution can artificially stabilize ICA curves and compress interannual variance, potentially masking genuine fluctuations in woodcock abundance. Furthermore, hunter-collected datasets rely on voluntary participation and self-reported effort, creating the potential for inconsistent data entry, recall bias in trip duration estimates, and selective reporting of successful outings. Hunters may be less likely to report low-yield trips, which would further upwardly bias abundance estimates.

The decadal analysis revealed two clear peaks in ICA values, occurring in late November and early February. These peaks correspond closely to the main post-nuptial and pre-nuptial migration periods described for woodcock in Europe [4,44] and in Italy [10,45]. These data are supported by monitoring studies showing consistent seasonal increases in woodcock presence in Italy during key migration periods, including late autumn and late winter–early spring, which are interpreted as phases of major migratory influx [45]. During these phases, large contingents of birds temporarily concentrate in suitable habitats, resulting in increased detectability and higher abundance indices. This is confirmed also by the weight trend: at the beginning of the season, when woodcocks arrive in Apulia during their post-breeding migration, they generally have lower average weights. The migration journey requires a lot of energy, and birds arrive at their wintering grounds underweight. In the American Woodcock, for example, the loss is approximately 10% of its overall weight [46]. As the season progresses, however, energy is recovered and bodyweight is gained.

The mean ICA curve across the three seasons showed a pronounced maximum in the first decade of February. However, this maximum is largely driven by the unusually high ICA values observed in early February 2024. This could be explained by the exceptionally mild winter that occurred in 2023/2024 in Italy, especially in February, with a +3.09 °C temperature anomaly [47], which likely contributed to an earlier onset of northward movements, a pattern that has also been observed in Sicily during the same season [48].

Taken together, across the three years studied, the temporal window during which the Apulian region experiences the bulk of pre-nuptial woodcock migration extends from the first decade of February to the first decade of March. Peak migration timing may vary between years in response to seasonal weather conditions, highlighting the influence of interannual climatic variability on migratory phenology. The mosaic of habitats available in Apulia—including woodlands, agricultural marginal areas, and scrub environments—provides suitable foraging and sheltering opportunities that are important for migratory woodcock at stopover sites. While direct tracking studies have revealed a wide range of individual migratory strategies and distances along the Italian peninsula, the consistent observation of woodcock throughout central and southern Italy underscores the region’s role as a significant component of the species’ migratory network [49].

Between these peaks, ICA values remained relatively stable, suggesting the presence of wintering birds that remain in the area for extended periods. This pattern is consistent with telemetry and ringing studies indicating partial winter site fidelity in Eurasian Woodcock, with some individuals remaining largely sedentary while others continue to move in response to weather conditions and habitat suitability [6,50,51]. However, slight oscillations in the ICA curves may also reflect opportunistic intra-seasonal movements. In southern Italy, and particularly in Apulia, these fluctuations could be related to so-called pendulum movements between southern Italy and the Balkan Peninsula, including Albania, especially when the latter is affected by severe cold spells. The occurrence of additional winter movements triggered by harsh weather conditions has been documented in other regions [52,53], demonstrating that Eurasian Woodcock may undertake supplementary winter displacements in response to freezing events. Although for the Balkans–Italy system this mechanism remains largely hypothetical and is inferred by analogy with observations from other countries, similar pendulum movements were already described in the 1970s in France between inland areas and coastal zones [54], supporting the plausibility of this interpretation.

Higher ICA values recorded during the post-hunting monitoring period are likely influenced not only by the absence of hunting and reduced disturbance but also by methodological differences between hunting and monitoring surveys. Comparable increases in abundance indices outside the hunting season have been reported elsewhere and should therefore be interpreted with caution, as they may partly reflect behavioural responses and effort-related effects rather than real increases in population size [38]. Hunting levels remained relatively stable across seasons, despite variation in hunting effort and trip duration. The lack of a clear decline in ICA values during the hunting period suggests that hunting mortality did not produce detectable short-term reductions in relative abundance at the regional scale. This pattern is consistent with previous findings indicating that, during migration peaks, local harvest losses are often compensated by the continuous arrival of new birds [4,41].

The decreasing trend observed in the Index of Mortality per Contact (IMC) may reflect changes in hunter behaviour, increased selectivity, or a higher proportion of non-lethal encounters. Similar trends have been interpreted in other studies as potential indicators of adaptive hunting strategies or changing hunter attitudes rather than changes in bird vulnerability alone [40].

Wing analysis showed a strong dominance of juveniles in the hunting bag, with young birds accounting for over 80% of hunted birds. This result is in accordance with the ten-year study carried out by the FIBec association that monitored the woodcock population in Italy from 2010 to 2019, where in south Italy a 79% age-ratio value calculated on 6283 samples was found [55,56]. Moreover, the result closely matches age ratios reported in numerous European studies, where juveniles typically represent the majority of the autumn and winter hunts [4,38]. In Italy, however, age-ratio is usually higher than in other European countries. This pattern is generally attributed to higher juvenile abundance following the breeding season and greater susceptibility of inexperienced birds to hunting [45]. It is important to acknowledge, however, that hunter behaviour itself may contribute to this demographic skew: hunters operating dogs in dense cover are more likely to encounter flushing birds, which are disproportionately juvenile. Adult birds, which tend to exhibit greater wariness and more cryptic behaviour, may be systematically underrepresented in the harvest regardless of their actual abundance. This selectivity limits the use of age ratios as unbiased proxies for breeding productivity, since the proportion of juveniles in the bag reflects both true cohort size and differential vulnerability. The relatively high proportion of late-hatched juveniles (jc4) is particularly noteworthy. Late-hatched birds have been associated with replacement clutches or second nesting attempts, which have been documented in woodcock under favourable environmental conditions or following early nesting failure [44,52]. The presence of a substantial jc4 component in the sample may therefore indicate a degree of reproductive flexibility that could contribute to population resilience.

The absence of significant differences in body mass between age classes is consistent with previous findings from wintering areas, where body condition tends to converge due to similar energetic demands and feeding opportunities [6,38]. These results suggest that, within the study area and period, hunting pressure does not appear to disproportionately affect specific demographic categories based on body condition. However, seasonal and geographical variation in body mass related to migration stage and weather severity has been reported elsewhere and may not be fully captured by the present dataset [51].

The present study confirms the usefulness of standardized hunter-based monitoring for assessing woodcock population trends at the regional scale, as also emphasized by previous European monitoring initiatives [4,19,41]. The apparent stability of abundance indices, combined with a harvest dominated by juveniles, suggests that current hunting pressure in the Apulia region does not cause immediate detectable impacts on wintering abundance. Nonetheless, the absence of independent validation through alternative survey methods—such as nocturnal point counts, playback surveys, or telemetry—means that the trends reported here cannot be cross-checked against unbiased population estimates. Without such validation, the magnitude and direction of residual biases in the ICA remain uncertain. Future monitoring efforts in the region should therefore aim to integrate at least a subset of independent, observer-based surveys to calibrate hunter-derived indices and improve the reliability of long-term trend assessments.

Woodcock populations are strongly influenced by large-scale factors such as breeding success, climatic conditions, and habitat availability along migration routes. As highlighted by several authors, local monitoring should therefore be integrated into coordinated, flyway-scale frameworks to effectively detect long-term population changes and support adaptive management [6,38]. Within such frameworks, hunter-based indices are best understood as relative measures of detectability under standardized effort rather than absolute population estimates, and their interpretation benefits greatly from cross-validation with independent data sources.

## 5. Conclusions

This study provides a comprehensive three-year assessment of Eurasian Woodcock (*Scolopax rusticola*) abundance, seasonal dynamics, and demographic structure in the Apulia region, based on standardized hunter-collected data. The use of the Index of Abundance per Trip (ICA), combined with detailed information on hunting effort and post-hunting monitoring, proved to be effective in describing temporal patterns in woodcock presence and abundance. Overall, ICA values remained stable across the three hunting seasons, indicating no evidence of marked short-term declines in regional woodcock abundance during the autumn–winter period. Clear seasonal increases in abundance were detected in late November and early February, corresponding to post-nuptial and pre-nuptial migration phases, respectively. These findings confirm the role of southern Italy as an important wintering and stopover area along the Mediterranean flyway.

Demographic analyses based on wing collections revealed that young birds account for more than 80% of hunted individuals, while the body mass did not differ significantly between age classes. The relatively high proportion of late-hatched juveniles suggests the possible occurrence of replacement or second nesting attempts, highlighting a degree of reproductive flexibility that may contribute to population resilience.

Taken together, the results suggest that current hunting pressure in the study area does not produce detectable short-term impacts on woodcock abundance at the regional scale. However, the strong influence of migratory fluxes on local abundance underscores the importance of interpreting regional trends within a broader flyway context.

The study highlights the value of standardized hunter-based monitoring as a cost-effective and reliable tool for the long-term population assessment of migratory game birds. Continuous data collection, integration with coordinated monitoring programs at national and international scales, and the extension of demographic sampling will be essential to detect long-term population changes and to support the adaptive and sustainable management of Eurasian Woodcock populations.

## Figures and Tables

**Table 1:** Hunting effort, number of observations, harvest, and abundance indices of woodcock for each hunting season.

	Hunting Seasons	2022/2023	2023/2024	2024/2025
Hunting period	Users	37	42	36
	Trips	501	646	648
	Hours	1802.5	2325.5	2.29
	Woodcocks observed	725	849	1.231
	Woodcock killed	253	272	349
	ICA ^1^	0.74	0.64	0.78
	IMC ^2^ (%)	34.9	32	28.35
	SdC	7 h 7 m	8 h 33 m	6 h 34 m
Monitoring period	Users	70	35	43
	Trips	381	186	218
	Hours	999	465	609.50
	Woodcocks observed	416	216	271
	ICA ^1^	0.93	0.98	0.97

^1^ ICA: Indice Cynégétique d’Abondance; ^2^ IMC: Hunting mortality rate.

**Table 2:** Statistical analysis of the two ICA series.

Parameter	Value
Pearson correlation coefficient (r)	0.998
r^2^	0.997
*p*-value	<0.0001
Covariance	1490.4
Sample size (*n*)	6
Statistic	34.571

**Table 3:** Woodcock data collected in the hunting seasons 2022/2023 and 2024/2025.

Hunting Seasons	2022/2023	2024/2025	Total
Wings collected (*n*)	277	437	714
Young (*n*)	233	361	594
Adult (*n*)	44	76	120
Age-ratio (%)	84.12	82.61	--
Adult body weight (g)	296.80	299.84	--
Young body weight (g)	296.54	301.43	--

## Data Availability

Data are contained within the Supplementary Materials.

## Supplementary Materials

The following supporting information can be downloaded at https://www.mdpi.com/article/10.3390/ani16101442/s1. S1: wing data; S2: ICA export 2024–2025; S3: ICA export 2023–2024; S4: ICA export 2022–2023.
